# Negative emotions and creativity among Chinese college students during the COVID-19 pandemic: the mediating role of psychological resilience and the moderating role of posttraumatic growth

**DOI:** 10.1186/s13690-022-00954-8

**Published:** 2022-08-22

**Authors:** Wei Zeng, Dongtao Huang, Qian Li, Yanhua Xu, Ziying Xu, Chujin Wu, Zhihao Chen, Yuhao Yang, Jinlian Shao, Xingrou Wu, Ziqi Zhu, Jiamin Wu, Yuqing Zeng

**Affiliations:** 1 School of Geography, South China Normal University, No. 55 West Zhong-Shan Avenue, Tianhe District, Guangzhou, 510631 China; 2 College of Resource Environment and Tourism, Capital Normal University, No.105 Xi-Sanhuan North Road, Beijing, 100048 China; 3 Office of International Cooperation and Exchange, Dongguan University of Technolog, No.1 University Road, Songshan Lake District, Dongguan, 523808 China

**Keywords:** Negative emotions, Creativity, Psychological resilience, Posttraumatic growth, Moderated mediation model, COVID-19

## Abstract

**Background:**

The aim of the study was to use a moderated mediation model to understand and examine the relationship between negative emotions and creativity among college students during the COVID-19 pandemic, using psychological resilience as a mediator and posttraumatic growth as a moderator.

**Methods:**

A sample of 881 college students in mainland China completed a self-report questionnaire that included four scales: the Depression-Anxiety-Stress Scale, Psychological Resilience Scale, Runco Ideational Behavior Scale and Posttraumatic Growth Inventory.

**Results:**

Findings indicated that:(1) negative emotions were a strong predictor of creativity; (2) psychological resilience partially mediated the association between negative emotions and creativity; and (3) posttraumatic growth moderated the positive effect of psychological resilience, such that the indirect effect between negative emotions and creativity via psychological resilience was stronger for someone with a low level of resilience.

**Conclusion:**

The findings further clarify the mechanisms that affect the relationship between negative emotions and creativity among college students.

## Background

The outbreak of the 2019 coronavirus disease (COVID-19) increased the occurrence of negative emotions among individuals. The outbreak was sudden, the disease was highly contagious, and this infectious respiratory disease rapidly became a global pandemic. In 2020, a total of 657,684 cases of infection were reported in Guangdong Province of China, with 1240 deaths. The epidemic has been not only a threat to life and physical health, but also has had an impact on mental health. Some people quickly became anxious, especially those quarantined because they had a probable COVID-19 infection or lived in a high-epidemic area [109].

In ordinary times, 17% of the adult population of the United States experience severe depression, but during the pandemic, the prevalence of depression and anxiety among Americans rose to 24.4 and 28.2%, respectively [95]. A similar trend has been observed in China. In 2019, the prevalence of anxiety among the Chinese population was 7.6% [46]. During the COVID-19 pandemic, however, 16.51% of the inhabitants of Hubei Province experienced such symptoms [110]. Another study of 1210 respondents in China showed that 84.7% had stayed at home 20–24 hours a day and experienced depression and anxiety that they reported as moderate (16.5%) to severe (28.8%) [98]. In conclusion, psychological problems, such as psychological distress, negative emotions and post-traumatic stress disorder, were caused by the varying degrees of injury or changes in life circumstances that people experienced during the epidemic. Therefore, it is important to study how people coped with psychological problems in this situation in order to remediate the psychological stress caused by the epidemic.

The impact of pandemic-related negative emotions on college students had attracted much attention. Henry and Crawford [42] proposed that depression, anxiety and stress could be combined into a higher-order psychological variable called *negative emotions*. Negative emotions refers to the negative impact on people’s psychology, causing some emotional fluctuations, resulting in functional disorders and behavioral disorders. Negative emotions generally include depression, anxiety and stress. These three dimensions — depression, anxiety and stress — are closely related to each other, which can better reflect the individual’s negative emotional experience. Facing the dual pressure of the pandemic and of academic work, college students were more likely to develop negative emotions. A study by Chen et al. [16] showed that, in the early stages of the pandemic, Chinese college students generally had four negative emotions: tension, anger, sadness and fear. A similar study [99] found that 56.04% of 3178 college students reported feelings of anxiety, and 28.48% may have experienced severe anxiety. According to these results, the COVID-19 pandemic has increased the occurrence of negative emotions among college students. If negative emotions are neglected for a long time, the individual can be under stress for a long time, which damages their physical and mental health [94].

Furthermore, the pandemic may have affected the development of college students’ creativity. In the 1950s, American psychologist J. P. Guilford introduced the term “creativity”. At present, most scholars believe that *creativity* is a developmental potential rather than an existing product, and it is also one of the most important qualities of human beings [7, 82]. Research has shown that creativity is closely related to the environment. From a macro perspective, broken-windows theory [68, 97], trait activation theory [86, 92], attention restorative theory [2] and other explanatory frameworks analyze the amplifying effect of environmental factors on innovation from different perspectives. From a micro perspective, individual characteristics are often stimulated and induced by environmental factors to affect individual creativity. For example, researchers found that external environmental conditions such as time pressure [8], task characteristics [24], task execution stage [50] and developmental feedback provided by the instructor [37] had a significant impact on creativity.

However, these studies were carried out in a laboratory environment and so the results cannot be extended to real-life situations. In fact, negative life events are widespread in real life, and groups affected by such an event often experience negative emotions [15, 34]. As a pandemic with a global impact, COVID-19 has undoubtedly aggravated the occurrence of negative emotions among college students who have been exposed to varying degrees of threat of injury or death. In the post-pandemic era, exploring how to stimulate and improve the creativity of college students will become not only a general theoretical problem but also a major practical problem that has to be solved.

According to the existing literature, the development of college students’ creativity is influenced in many ways, and emotional problems caused by environmental factors may be important among these. A series of related theories have been developed, such as emotional arousal theory [48], emotional consistency retrieval theory [29], and mood-repair theory [1]. A small number of studies have explored the relationship between negative emotions and the creativity of college students [23, 85]. Recent research has pointed out that the relationship between emotion and creativity is not static [52] and that there might be an important intermediary variable between the two. Psychological resilience has frequently been found to be related to creativity [100]; however, few studies focused on the relationship between psychological resilience and creativity, and the possible mediating role of psychological resilience between negative emotions and creativity. Another variable, posttraumatic growth, may be related to psychological resilience [59, 108]. Again, few studies have focused on the moderating effect of posttraumatic growth on psychological resilience as it mediates between negative emotions and creativity.

In order to deepen the understanding of the development of the creativity of college students during the COVID-19 pandemic, we explored the relationship between negative emotions and creativity, as well as the mediating role of psychological resilience and the moderating role of posttraumatic growth. In the next section, the definition of these four variables and the relationship between them will be introduced.

### The relationship between negative emotions and creativity

In the evolution of creativity research, early studies mainly focused on individuals such as Aristotle, Leonardo da Vinci, and Newton, who had a long history of talent or genius [89]. In the eighteenth century, creativity was considered to be a genius or talent [4], and it was not until the 1950s that American psychologist J. P. Guilford proposed the term *creativity* [81]. With the advancement of science and technology and the development of psychology, scholars have been increasingly studying the nature of creativity, and its definition has been in continuous evolution. The present study is based on Runco and Basadur’s [83] conceptualization of creativity.

According to the existing literature, researchers have concentrated on analyzing the direct impact of emotion on creativity but have neglected to explore the mechanism of emotion in depth [68]. Emotions can generally be categorized as negative, positive, or neutral. To date, research on the influence of emotion on creativity has mainly focused on its positive and negative dimensions [49]. Certain researchers have concluded that positive emotions promote flexibility in thinking, thus enhancing creativity, while negative emotions narrow the scope of cognition and inhibit creativity [6, 12, 39]. A possible explanation for these conclusions may be that positive emotions can promote the secretion of dopamine in the brain, which in turn regulates its cognitive function and promotes creativity [43, 44]. Other researchers believe that both positive and negative emotions can promote creativity, but that they each act on different components of creativity [25, 37]. Furthermore, most existing research has been carried out in developed countries and based on specific tasks completed in a laboratory, which may have obscured the essential relationship between emotion and creativity.

Negative emotions have a negative impact, causing some emotional fluctuations and resulting in functional and behavioral disorders [18]. They are general emotional dimensions that reflect subjective stressful experience and unpleasant engagement. Many researchers have concluded that negative emotions can have a positive effect on creativity [3, 25, 28, 48]. When Russ and Grossman-McKee [85] asked research subjects to create collages with materials of different shapes, sizes and colors on cardboard, those experiencing negative emotions were more creative. Further, Vosburg and Kaufmann (as cited in [47]) found that participants with negative emotions performed best in the insight experiment, suggesting that negative emotions can improve integrative thinking. Damian and Robins [23] found that negative emotional states increased an individual’s level of self-esteem, thereby improving their performance in creative tasks. From a cognitive perspective, some researchers have suggested negative emotions improve creativity by improving cognitive persistence [51, 73]. Furthermore, creativity can be affected by different cognitive styles, and impulsive cognitive styles are more conducive to creativity [5].

Negative emotions can affect creativity in other ways. In fact, appropriate negative emotions help people better cope with the problems of life, enhancing their ability to resist stress and thereby promoting creativity. Although it has been shown that different negative emotions affect creativity differently [52, 75], the present study will follow Henry and Crawford [42] in considering depression, anxiety and stress to be negative emotions. Therefore, the following hypothesis is proposed:

- H1: Negative emotions have a positive predictive effect on creativity.

### The mediating role of psychological resilience

*Psychological resilience* refers to an individual’s ability to adapt and to develop normally after encountering illness, frustration, trauma, adversity or another major stress environment [105]. This stable psychological quality enables an individual to maintain mental health and a happy life when faced with various pressures and to recover from stress, danger, and other adversities. Individuals with higher levels of resilience attain better mental-health outcomes (such as with regard to depression, anxiety and post-traumatic stress disorder) following life adversity and major threats [72]. Tugade et al. [93] found that individuals with high psychological resilience were good at using positive emotions such as humor and creative exploration to effectively deal with adversity. Connor and Davidson [21] described psychological resilience as a cognitive processing method that negates the influence of negative mood disorders, and so it was an important goal in the treatment of anxiety, depression and stress response. In addition, they showed that psychological resilience could be changed and improved with treatment. In summary, persons with high psychological resilience are more likely to mobilize their inner strength after experiencing negative life events.

College students are in a life stage characterized by rapid physical and mental development, and only with high psychological resilience can they cope with negative emotions in daily life. Du et al. [26] found a significant negative correlation between employment anxiety and the psychological resilience of Chinese college students. Yu et al. [107] focused on a special group of college students, Chinese army recruits who had just finished a three-month recruit training program, finding that resilience and positive/negative coping partially mediated the effect of anxiety on perceived stress-related growth.

Another even more special group exhibited a similar pattern: Galatzer-Levy et al. [31] studied police officers who worked in high-risk circumstances and found that lower levels of negative emotions and higher levels of positive emotions predicted higher psychological resilience. To date, most research studies have focused on a single variable—depression or anxiety—seldom addressing the crucial factor of stress. Research on the relationship between multi-dimensional indicators of negative emotions and psychological resilience is lacking. Therefore, the following hypothesis is proposed:

- H2a: Negative emotions have a negative predictive effect on psychological resilience.

Psychological resilience plays an important role in the creativity of college students. In fact, the process of how psychological resilience affects creativity is complex. Only a few empirical studies have investigated the influence and mechanism of psychological resilience on college students’ creativity. For example, Li et al. [55] showed that nursing undergraduates with a high level of creativity had relatively high psychological resilience. The results of this study support the conclusion that higher psychological resilience promotes creativity or innovative behavior. Therefore, the following hypothesis is proposed:

- H2b: Psychological resilience has a positive predictive effect on creativity.

Negative emotions can make individuals strive to change the status quo, which causes more mood swings, which is conducive to identifying the most creative plan from a variety of possibilities. Bledow et al. [12] showed that when individuals experience negative emotions for a period of time and then leave them behind and enter a positive emotional state, this dynamic process brings about a high level of creativity. Thus, if a college student enters a negative emotional state, and their psychological resilience is activated, the influence of negative emotions may be alleviated, which leads to changes in their creativity.

On the basis of the literature and the above three hypotheses, it is evident that negative emotions, psychological resilience, and creativity are closely related, but no research has specifically explored the relationship between the three. Therefore, the following hypothesis is proposed:

- H2: Psychological resilience mediates negative emotions and creativity.

### The moderating role of posttraumatic growth

Tedeschi and Calhoun [90] scientifically measured psychological growth after experiencing trauma, proposing the term *posttraumatic growth*. They (2004) continued to explore the phenomenon, characterizing it as changes in the perception of self, in the experience of relationships with others and in general philosophy of life. We have adopted heir point of view, and so posttraumatic growth is presented as a positive psychological change achieved by individuals through active resistance after encountering a traumatic event [90]. The process of achieving this state can improve the individual’s psychological condition and reduce their negative psychological experience. Chi et al. [20] showed that, during the COVID-19 pandemic, Chinese university students demonstrated an ability to effectively cope with challenges and to experience posttraumatic growth.

It is inevitable that an individual will encounter or witness negative life events. Post-traumatic stress disorder caused by such events is often accompanied by negative emotions such as anxiety, depression and fear. The correlation between the two had attracted the attention of researchers [64, 94]. In turn, a person with post-traumatic stress disorder may experience negative emotions such as anxiety and depression and be more likely to commit suicide [76].

Posttraumatic growth is the positive side of experiencing traumatic events, the positive impact of a traumatic event on an individual. Such a change is related to growth-related personal coping strategies and mental health [10, 88]. A number of studies have found that emotional expression and emotional processing predict the psychological growth level of persons who have been traumatized: those with a high level of positive emotion were more likely to grow from trauma [62, 70]. Conversely, people with high levels of negative emotions did not easily achieve high levels of energy and growth after trauma. A small number of longitudinal studies of negative emotions and posttraumatic growth have occurred, but these did not clarify the relationship between the two phenomena. Therefore, this research proposed the following hypotheses:

- H3a: Negative emotions have a negative predictive effect on posttraumatic growth.

Positive responses to negative life events are an important factor in successful recovery and readaptation, and they are also a prerequisite for posttraumatic growth. Experiencing negative emotions after encountering major disasters, accidents and setbacks are very common and cannot be prevented. How to actively respond is the key. The individual’s ability to do this is related to their personality traits and coping styles [77]. Active coping strategies alleviate individual trauma, facilitate readaptation after trauma and significantly predict the occurrence of posttraumatic growth [106].

Based on the previous discussion, it can be concluded that posttraumatic growth represents a positive psychological change [107], so it can be conjectured that an individual’s level of resilience can affect the degree of posttraumatic growth. In fact, researchers have found that psychological resilience has a positive effect on posttraumatic growth [59, 108]. In the medical field, doctors often help patients improve their psychological resilience to facilitate posttraumatic growth [17, 67]. It seems obvious that, in a long-term, dynamic process of growth and development, people with high resilience have more cognitive flexibility and can adjust their cognition more flexibly to achieve growth after encountering a traumatic event. In other words, posttraumatic growth is an important influencing factor on psychological resilience [60, 91]. Therefore, the following hypothesis is proposed:

- H3b: Psychological resilience has a positive predictive effect on posttraumatic growth.

Individuals differ in how they experience negative life events. On the one hand, negative emotions resulting from negative life events may inhibit individual creativity [11]. On the other hand, such events can also become an opportunity for growth [103]. If an accidental injury enhances the development of individual talent and creative performance, it is likely to improve learning and life. For example, Lin and Liu [57] studied the internal mechanism of the posttraumatic growth of Chinese college students who had failed to start a business, finding that the trauma of business failure induced change in their entrepreneurial attitudes and personality factors, which in turn triggered change in their entrepreneurial behavior. Obviously, these individuals used their negative experiences as a source of inspiration and motivation. In Forgeard’s view [30], creativity constitutes a way of expressing posttraumatic growth. However, the literature contains few examples of discussion of the relationship between posttraumatic growth and creativity, and empirical evidence for the consequences of posttraumatic growth are uncertain [38]. The following hypothesis is proposed:

- H3c: Posttraumatic growth has a positive predictive effect on creativity.

Posttraumatic growth strengthens an individual’s psychological resilience, allowing the setting of new goals in the event of failure and the turning of disadvantages into opportunities and challenges. Chen and Padilla [19] found that positive emotional experiences enabled language learners to strengthen their psychological resources and to perceive the learning environment from a creative and flexible perspective. In other words, psychological resilience contributes to the generation of creativity, and it strengthens posttraumatic growth. Therefore, the following hypothesis is proposed:

- H3d: Posttruaumatic growth moderates the predictive effect of psychological resilience on creativity.

For college students, the research on how posttraumatic growth plays a positive role in creativity is worth exploring. The COVID-19 pandemic has been a global negative life event, and has increased the occurrence of negative emotions among individuals. If college students can emerge from the pandemic, perhaps their creativity will improve. Through the previous discussion, it can be seen that negative emotions may be more conducive to the creative process, and psychological resilience is the pathway that connects the two phenomena. If psychological resilience is further affected by posttraumatic growth, can it affect creativity? On the basis of the literature review and the hypotheses proposed above, it is evident that negative emotions, psychological resilience, posttraumatic growth and creativity are closely connected. However, no study has yet explored the relationship between the four. Therefore, the following hypothesis is proposed:

- H3: Posttraumatic growth plays a moderating role on the second half of the intermediary path proposed in H2.

**Fig. 1:**
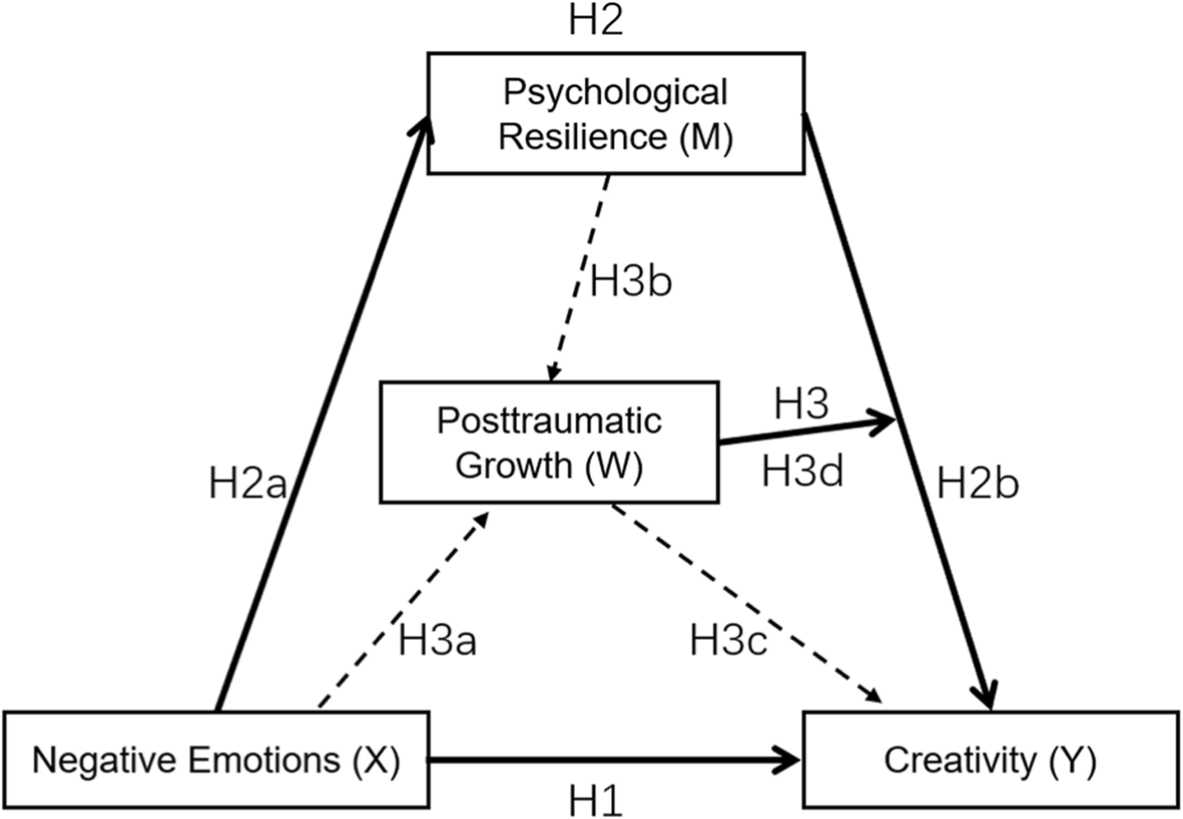
Conceptual model of the relationships between negative emotions, creativity, psychological resilience and posttraumatic growth

In summary, based on the relationship of four variables above, we propose a moderator-mediator model (see Fig. 1). Our aim is to provide insight into how (the mediating role of psychological resilience) and under what circumstances (the mediating role of posttraumatic growth) negative emotions lead to creativity.

How to promote creative talent has always been a major issue of concern to all countries. College or university is a key stage for cultivating high-end talent. Developed countries regard cultivating creativity in college students as an important national strategy. This study aims to deepen understanding of creativity and to suggest new ways of cultivating the creativity of college students.

We introduces two innovations in this study. First, depression, anxiety, and stress have been unified into a single variable—negative emotions. Second, data were collected from a large sample in a real-life environment because all participants would inevitably have experienced COVID-19 and living through the epidemic. Negative emotions, posttraumatic growth and psychological resilience all play an important role in the cultivation of creativity, but the mechanism of these factors with regard to the creativity of college students is still unclear. Therefore, we constructed a moderated mediation model to examine the impact of negative emotions on the creativity of college students during the COVID-19 pandemic, along with the mediating role of psychological resilience and the moderating role of posttraumatic growth.

## Methods

### Participants and procedures

This research was conducted at a college of technology in Guangdong Province, China. This college has 13 teaching units, offers 44 undergraduate majors, and enrolls more than 15,500 full-time students. In order to better understand the emotional characteristics and psychological states of potential respondents, we conducted exploratory focus-group interviews with 12 students at the college before finalizing the research design. By doing so, we previewed what the students would report. The majority of interviewees indicated that they were experiencing negative emotions during the COVID-19 outbreak, so that the epidemic was actually having a significant impact on their studies and on their lives.

For the study, stratified random sampling was used: all students were stratified by department and then randomly sampled. A total of 918 students completed an online questionnaire. The exclusion of those who did not live in Guangdong Province when they completed the questionnaire led to an actual valid sample of 881. All respondents, 317 males (35.982%) and 564 females (64.018%), were from the same grade.

The questionnaire was available from April 10 to June 15, 2020. While students enrolled in same-level English classes took a break, a researcher explained the purpose and significance of the research and invited them to participate. In China, QR codes are usually used to access web functions with a smartphone, such as making payments, registering information, and accessing specific web pages. For this study, interested students scanned the QR code that linked to the webpage where the questionnaire was located, which greatly improved the efficiency of data collection.

### Instruments

Data were collected by means of a survey questionnaire that consisted of five sections: demographic information and four scales. Demographic statistics included gender and place of current residence; The scales were the Depression-Anxiety-Stress Scale 21, Psychological Resilience Scale, Runco Ideational Behavior Scale and Posttraumatic Growth Inventory. Three scales initially developed in English were translated into Chinese for the purposes of this study and then tested using back-translation [14]. Inequivalences were resolved before data collection.

#### Depression-anxiety-stress scale 21

We used Depression-Anxiety-Stress Scale 21 revised by Antony, which has 21 items divided into three dimensions: depression, anxiety, and stress [58]. The scale is a set of self-rated statements by which adult respondents assess depression, anxiety and stress. The scope of its application has been extended to children, adolescents and the elderly, and it is used in clinical practice. The items are rated on a 4-point Likert scale. The Cronbach’s alpha of the scale in this study was 0.960.

#### Psychological resilience scale

We used Psychological Resilience Scale, created by Hu and Gan [45] after reviewing relevant Chinese research, along with that of Connor and Davidson [21]. The scale has a total of 27 items, divided into five dimensions: goal focus, emotional control, positive cognition, family support, and interpersonal assistance The items are rated on a 5-point Likert scale. The Cronbach’s alpha coefficient of the scale in this study was 0.860.

#### Runco ideational behavior scale

We used the conceptual behavior scale proposed by Runco et al. [84]. The scale had a total of 23 items that measure creative behavior. As a kind of creative achievement test, the scale focuses on the level of individual self-assessment of creative behavior from the perspective of daily life. The items are rated on a 5-point Likert scale. The Cronbach’s alpha coefficient of the scale in this study was 0.938.

#### Posttraumatic growth inventory

We used the Chinese version of the Posttraumatic Growth Scale, originally developed by Tedeschi and Calhoun [90] and translated and adapted by Geng et al. [35] to suit the Chinese context. The scale has a total of 21 items, divided into five dimensions: interpersonal relationships, new possibilities, personal strength, spiritual changes, and appreciation of life. The items are ated on a 6-point Likert scale. The Cronbach’s alpha coefficient of the scale in this study was 0.958.

### Data analysis

This study used self-assessment questionnaires to collect data, and it is possible that the results were affected by common method deviations. Following the suggestions of Zhou and Long [111], the data-collection procedure was controlled and conducted anonymously, and some items were designed to be reverse-scored. In order to ensure the validity of data analysis, the data collected were tested for common method bias using the Harman single factor test [74]. We conducted this test on all 92 items in the questionnaire related to the four variables. Generally speaking, the variance explained by the first factor needs to be less than 40%, which is the cutoff value for dividing the deviation of the common method [54].

After testing for common method bias, we performed descriptive statistical analysis. We used SPSS23.0 software to calculate the mean and standard deviation of each variable, observed the change trend and dispersion of variables, and calculated the Pearson correlation coefficient to test the correlation between each variable.

Finally, we used the PROCESS program (version 3.3), which was developed by Hayes [41], to test the mediating effect of psychological resilience and the moderating effect of posttraumatic growth. This software program can handle complex models such as regulation, mediation, and mediation with regulation. All bootstrapping analyses in this study used 5000 repeated sampling to construct a 95% deviation correction confidence interval (CI). If zero is not included between the lower and upper limits of the CI, then the corresponding effect is significant [87].

## Results

### Testing for common method Bias

Using principal component factor analysis, we identified 15 factors with eigenvalues greater than 1, and the first factor obtained accounted for only 21.260% of the variance. The first factor obtained after using the maximum-variance method was 14.733%, which is less than the critical value of 40% and indicates the absence of serious common method bias in this study.

### Descriptive statistics and correlations analyses

We examined the relationships among the four variables, and these s are presented in Table 1. The results showed that negative emotions were weakly and positively correlated with creativity (*r* = 0.068, *p* < 0.05) and negatively correlated with resilience (*r* = − 0.496, *p* < 0.01) and posttraumatic growth (*r* = − 0.081, *p* < 0.05). Creativity was positively correlated with psychological resilience (*r* = 0.198, *p* < 0.01) and posttraumatic growth (*r* = 0.434, *p* < 0.01). Psychological resilience was positively correlated with posttraumatic growth (*r* = 0.414, *p* < 0.01).

**Table 1:** Means, standard deviations and correlations between variables (*N* = 881)

Variables	M	SD	1	2	3	4
1. Negative emotions	1.626	0.562	1			
2. Creativity	3.249	0.563	0.068*	1		
3. Psychological resilience	3.417	0.443	−0.496**	0.198**	1	
4. Posttraumatic growth	3.301	1.003	−0.081*	0.434**	0.414**	1

*p < 0.05, **p<0.01

### Mediation analysis

The study used Model 4 of the SPSS PROCESS component to perform multiple regression analysis. The bootstrap method was used to test the CI estimation, resampling 5000 times and calculating the 95% CI. In Model 1, negative emotions positively predicted creativity (β = − 0.068, *t* = 2.010, *p* < 0.05). In Model 2, negative emotions negatively predicted psychological resilience (β = − 0.391, *t* = − 16.936, *p* < 0.001). In Model 3, negative emotions (β = 0.220, *t* = 5.887, *p* < 0.001) and psychological resilience (β = 0.390, *t* = 8.218, *p* < 0.001) both positively predicted creativity.

Regarding the determination of the mediation effect, we adopted the current mainstream view, using the bootstrap method to analyze whether the product term of the regression coefficient from the independent variable to the mediator variable and the regression coefficient from the mediator to the dependent variable were significant and did not contain 0 [87]. The test result showed that the total effect of negative emotions on creativity was 0.068 (95% CI = [0.002, 0.134]), direct effect (95% CI = [0.147, 0.294]) and indirect effect (95% CI = [− 0.196, − 0.111]) and did not contain 0. In summary, the partial mediating equation model of psychological resilience was confirmed (Table 2).

**Table 2:** Testing the mediation effect of psychological resilience on creativity (*N* = 881)

Predictors	Model 1: CT			Model 2: PR			Model 3: CT		
	β	SE	*t*	β	SE	*t*	β	SE	*t*
NE	0.068	0.034	2.010*	−0.391	0.023	−16.936***	0.220	0.037	5.887***
PR							0.390	0.047	8.218***
*R*^*2*^					0.246			0.076	
*F*		4.042*			286.836***			35.942***	

*CT* Creativity, *NE* Negative emotions, *PR* Psychological resilience

*p < 0.05, ***p < 0.001 (2-tailed)

### Moderated mediation test

According to the hypothesis, the adjustment variable—posttraumatic growth—was added to the second half of the mediation model, and Model 14 was used to analyze the model. In Model 2, the product of psychological resilience and posttraumatic growth was statistically significant in predicting creativity (β = − 0.085, t = − 2.544, *p*<0.001), which shows that posttraumatic growth could play a moderating role in predicting creativity by means of psychological resilience. The results are presented in Table 3.

**Table 3:** Moderated mediation test (*N* = 881)

Predictors	Model 1: PR			Model 2: CT		
	β	SE	*t*	β	SE	*t*
NE	−0.391	0.023	−16.936***	0.154	0.035	4.393***
PG				0.139	0.049	2.852**
PR				0.230	0.019	12.209***
PR * PG				−0.085	0.033	−2.544***
*R*^*2*^		0.246			0.212	
*F*		286.836***			58.799***	

*NE* Negative emotions, *CT* Creativity, *PR* Psychological resilience, *PG* Posttraumatic growth

**p < 0.01, ***p < 0.001 (2-tailed)

In order to better explain the moderated mediation model, we divided scores for psychological resilience nto three groups of high, medium, and low (plus or minus 1 standard deviation). A simple slope test was used to investigate the role of posttraumatic growth, and it showed that the mediating effect of posttraumatic growth in the relationship between psychological resilience and creativity gradually increased from the low group to the middle group. The results are shown in Table 4. However, when the posttraumatic growth was positive by 1 standard deviation, the 95% bootstrap CI contained 0, indicating that the mediating effect was not significant.

**Table 4:** Posttraumatic growth moderates the relationship between psychological resilience and creativity (*N* = 881)

Variables	Effect	SE	95%CI	
			LLCI	ULCI
Low(*M*-1 *SD*)	−0.088	0.026	−0.139	− 0.039
Medium(*M*)	−0.054	0.020	−0.095	− 0.016
High(*M* + 1 *SD*)	−0.021	0.026	−0.072	0.029

## Discussion of the results

The results presented above confirm the hypotheses proposed and the results of previous research.

First, the results are consistent with H1 and also with the results of other studies. We found a weak and positive predictive relationship between negative emotions and creativity [36, 83, 102]. In our preliminary focus-group interviews, we found that the outbreak was leading to negative emotions among college students. They needed to form new learning habits to succeed in their professional courses because they were encountering difficulties in completing academic tasks while learning online. They perceived a large gap between their current environment and their ideal state, and they wanted to work hard to change the status quo [96]. From a neuropsychological point of view, this was due to the increased secretion of serotonin caused by negative emotions, which prompted them to work harder to change their circumstances [25]. Another possible explanation is that negative emotions helped create a strong sense of self-reflection and resilient thinking in individuals [67], stimulating them to explore the real environment [28], and thus enhanced creativity. In general, negative emotions can promote individual motivation, make individuals persist in tasks longer and devote more energy to them, which may increase creativity.

Second, the study results are also consistent with H2a, H2b and the findings of other studies. Negative emotions have a negative predictive effect on psychological resilience [13], which is consistent with the findings of Morote et al. [66] that strong psychological resilience predicts that fewer negative emotions are experienced. The hierarchical model theory of psychological resilience states that individuals with high psychological resilience actively and effectively mobilize resources to deal flexibly with stress and avoid continuing to fall into negative emotional states [71]. In the medical field, interventions with regard to psychological resilience have helped reduce individual mental-health problems [43], and physicians have come to pay more attention to psychological resilience and emotional changes in their patients [33]. Furthermore, psychological resilience has a positive predictive effect on creativity. Runco [80] discussed the close relationship of creativity to adversity. Psychological resilience has even been named as one of the manifestations of creative thinking [65], and Barron’s [9] definition of creativity echoed the definition of psychological resilience. Individuals with high psychological resilience can quickly mobilize sufficient resources to maintain a balanced state in the face of uncertain environments. Even in the event of failure or setback, psychological resilience makes it possible to recover quickly from adversity and continue to invest in innovative work, and creative participation in turn can strengthen psychological resilience [63].

Third, these results confirm H2 and the findings of previous research. The psychological resilience of participating college students played a partial and positive mediating role between negative emotions and creativity. When encountering a negative life event, if an individual does not have sufficient resources to alleviate the adverse effects of the event, emotional problems can easily arise. If this occurs, psychological resilience can act as a protective factor, effectively offsetting the negative effects of negative emotions and helping them respond in a positive way [5]. In other words, the stronger the psychological resilience of an individuals the more willing they are to regulate their negative emotions by themselves and to keep away from stressors through active and creative participation [22].

Fourth, the research results are also consistent with H3a, H3b, H3c, and H3d and with the findings of other studies. To begin, the negative emotions of participating college students had a negative predictive effect on posttraumatic growth. Consistent with the findings of Ma et al. [61], in the early stages of a trauma, negative emotions can lead to posttraumatic symptoms that arouse negative emotions and constrain an individual’s cognitive evaluation of the event, ultimately preventing posttraumatic growth [78]. Furthermore, the psychological resilience of participating college students had a positive predictive effect on their posttraumatic growth, which is consistent with the findings of Nishi et al. [69], Westphal and Bonanno [101], and Duan et al. [27]. They found that individuals with strong resilience are more likely to focus on their connections with others, discover new possibilities in life, appreciate life or feel enhanced after experiencing trauma. Moreover, the posttraumatic growth of participating college students had a positive predictive effect on creativity among the respondents. Consistent with the findings of Liang et al. [56], growth after experiencing adversity can make individuals more creative. For example, for the average person, creativity is higher among adolescents who have experienced loss of parents at an early age, survived a bushfire, or survived an earthquake [40, 56, 79]. In addition, an increase in creative thinking may be an important manifestation of posttraumatic growth in the cognitive process.

Fifth, the research results confirm H3; that is, the posttraumatic growth of college students plays a regulatory role in the second half of the mediating path of negative emotions and creativity. According to the above literature discussion, previous studies on the influence of external environmental conditions and negative emotions on creativity were mostly carried out in specific laboratory conditions in developed countries, and the research results may be biased. However, the COVID-19 pandemic has created negative living environments around the world. It enables this study to investigate the relationship between negative emotions, resilience, post-traumatic growth and creativity by conducting a large data sample survey in real life situations. Since all participants inevitably experienced and grew up with COVID-19, the results of this study may be more relevant. To begin, positive emotions are the result of cognitive adjustment, while negative emotions are often the catalyst for such adjustment. Typically, experimental tasks associated with creativity research are generally cognitive in nature and of short duration. Furthermore, different levels of psychological resilience affect creativity differently, which is consistent with the results of previous studies. The lower level of resilience of uninjured individuals relative to the injured population has a somewhat greater impact on their posttraumatic growth. Positive attitudes toward negative life events among college students with low levels of resilience are more likely to have a positive effect on creativity. One study investigated the relationship between mental resilience and mental health of Wuhan high school students during the epidemic and concluded that mental resilience on emotion regulation could protect the mental health of Wuhan high school students [104]. Finally, the processing of posttraumatic cognition is a necessary process of post-traumatic growth. In the early posttraumatic cognitive processing of trauma, negative emotions play a very important role. Meanwhile, negative emotions and posttraumatic growth are affected by time factors [32, 53].. Therefore, in real life, after a longer period in a negative emotional state, through the emotional regulating effect of resilience that can change negative emotions into positive emotions, college students are often able to overcome the trauma and grow. They can also further enhance their resilience, which eventually acts on their creativity.

### Implications

In a theoretical sense, the results link the negative emotions experienced by college students during the COVID-19 pandemic to creativity, deepening understanding of the effect of such emotions on the creative process. The analyses of mediation and moderation effects revealed that helping college students recover from trauma is helpful to improve their psychological toughness and creativity.

In a practical sense, the proposed relationship between the four variables may help learning managers more effectively foster the creativity of college students by paying more attention to their mental health and by helping them overcome adversity and increase their psychological resilience. For example, teachers can provide students with opportunities to participate in meaningful activities and establish and maintain high expectations. Furthermore, school administrators can construct a variety of support systems for students instead of relying solely on external incentives or ideological and political education.

### Limitations and future directions

This study has certain shortcomings, which also provide direction for further exploration during follow-up research. First, although the purpose of the study was to examine the impact of negative emotions on the creativity of college students and the associated mechanism, due to the limitations of data collection, the influence of gender, age and major subject and other variables on the relationship was not controlled. Second, we adopted a cross-sectional design. Although a cross-sectional study combined with data analysis can explore the relationship between different variables, the causal relationship between variables has not been fully revealed. Third, due to the complexity of emotion and creativity, research results often differ. Negative emotions in this study were defined as depression, anxiety and stress, but negative emotions also include tension, loneliness, anger and others.

## Conclusions

We constructed a moderated mediation model to investigate the impact of negative emotions on the creativity of college students during the COVID-19 pandemic. The results showed that (1) negative emotions were a strong predictor of creativity; (2) psychological resilience partially mediated the association between negative emotions and creativity; (3) posttraumatic growth moderated the positive effect of psychological resilience such that the indirect effect of negative emotions on creativity via psychological resilience was stronger for an individual with a low level of resilience. The findings further clarify the mechanisms that affect the relationship between negative emotions and creativity among college students.

## Data Availability

The datasets used and/or analyzed during the current study are available from the corresponding author on reasonable request.

## Abbreviations

NE: Negative emotions
CT: Creativity
PR: Psychological resilience
PT: Posttraumatic growth
